# Synthesis and Gas-Permeation Characterization of a Novel High-Surface Area Polyamide Derived from 1,3,6,8-Tetramethyl-2,7-diaminotriptycene: Towards Polyamides of Intrinsic Microporosity (PIM-PAs)

**DOI:** 10.3390/polym11020361

**Published:** 2019-02-19

**Authors:** Giuseppe Genduso, Bader S. Ghanem, Yingge Wang, Ingo Pinnau

**Affiliations:** 1 Functional Polymer Membranes Group, Advanced Membranes and Porous Materials Center, Division of Physical Sciences and Engineering, King Abdullah University of Science and Technology, Thuwal 23955-6900, Saudi Arabia; giuseppe.genduso@kaust.edu.sa (G.G.); bader.ghanem@kaust.edu.sa (B.S.G.); 2 Advanced Membranes, and Porous Materials Center, Division of Physical Sciences and Engineering, King Abdullah University of Science and Technology, Thuwal 23955-6900, Saudi Arabia; yingge.wang@kaust.edu.sa

**Keywords:** triptycene diamine, glassy polymers, polyamides, PIM-polyamides, gas separation, mixed-gas permeation, gas sorption, intrinsic microporosity

## Abstract

A triptycene-based diamine, 1,3,6,8-tetramethyl-2,7-diamino-triptycene (TMDAT), was used for the synthesis of a novel solution-processable polyamide obtained via polycondensation reaction with 4,4′-(hexafluoroisopropylidene)bis(benzoic acid) (6FBBA). Molecular simulations confirmed that the tetrasubstitution with *ortho*-methyl groups in the triptycene building block reduced rotations around the C–N bond of the amide group leading to enhanced fractional free volume. Based on N_2_ sorption at 77 K, 6FBBA-TMDAT revealed microporosity with a Brunauer–Emmett–Teller (BET) surface area of 396 m^2^ g^−1^; to date, this is the highest value reported for a linear polyamide. The aged 6FBBA-TMDAT sample showed moderate pure-gas permeabilities (e.g., 198 barrer for H_2_, ~109 for CO_2_, and ~25 for O_2_) and permselectivities (e.g., αH_2_/CH_4_ of ~50) that position this polyamide close to the 2008 H_2_/CH_4_ and H_2_/N_2_ upper bounds. CO_2_–CH_4_ mixed-gas permeability experiments at 35 °C demonstrated poor plasticization resistance; mixed-gas permselectivity negatively deviated from the pure-gas values likely, due to the enhancement of CH_4_ diffusion induced by mixing effects.

## 1. Introduction

Polyamides are synthetic macromolecules containing amide groups (–CO–NH–) mainly prepared via polycondensation reaction between diamines and diacid-type monomers. Aromatic and semi-aromatic polyamides have been broadly applied in the industry, due to their extraordinary mechanical, chemical, and thermal properties [1]. For example, aromatic polyamides (“aramids”) are in use for the production of synthetic fibers of superior mechanical strength and heat resistance (e.g., Kevlar and Nomex fibers). Another important application of aromatic polyamides is the production of thin-film composite membranes by interfacial polymerization [2] for water desalination via reverse osmosis (RO); the employment of these membranes revolutionized desalination industry by making RO more cost efficient than conventional energy-intensive thermal processes [3].

Polyamides can be synthesized as fully-crosslinked networks producing surface areas exceeding 500 m^2^ g^−1^ [4,5,6]. For gas adsorption and gas storage applications, aromatic network polyamides are competitive with polymers of intrinsic microporosity (PIMs) [7,8,9], metal organic frameworks (MOFs), covalent organic frameworks (COFs) [10], porous poly(aryleneethynylene) (PAE) [11], porous aromatic frameworks (PAFs) [12], conjugated microporous polymers (CMPs) [13], and hypercross-linked polymers (HCPs) [14]. Although crosslinked aromatic network polyamides can exhibit high surface areas, they are insoluble and, therefore, cannot be utilized to synthesize integral asymmetric and thin-film composite membranes for gas separations. 

Linear, uncrosslinked aromatic polyamides are solution-processable; however, these materials generally display low fractional free volume (generally between 0.14–0.16 [15]) because interchain hydrogen bonding induces tight chain packing. Hence, gas permeabilities of linear aromatic polyamides are normally lower than those of polycarbonates, polysulfones, polyimides, and other conventional low-free-volume glassy polymers [15,16]. On the other hand, gas-pair permselectivities of linear polyamides (particularly for hydrogen applications) are notable; this has led to structural modifications to enhance the gas permeability of linear polyamides [16,17,18,19,20,21,22,23,24,25,26,27,28,29,30,31,32,33]. 

Very high gas permeabilities and moderate gas-pair selectivities are displayed by linear PIMs, such as ladder PIMs (e.g., PIM-1) and PIM-PIs (polyimides of intrinsic microporosity) that have attracted significant interest for membrane applications in the past decade [8,34,35,36,37]. More recently, the *fine-tuning* of the sites of contortion of these polymers has strongly improved the performance of these materials establishing the 2015 Robeson upper bound for O_2_/N_2_, H_2_/N_2_, and H_2_/CH_4_ separations [38]. Moreover, by appropriate selection of functional groups (e.g., –OH, –COOH) PIM-PIs have also defined the recently introduced 2018 mixed-gas upper bound for CO_2_/CH_4_ separation [37].

Likewise, the inclusion of bulky contortion sites—e.g., spirobifluorene, ethanoanthracene, Tröger’s base-, or triptycene-based moieties—in the repeat unit of aromatic polyamides [25,39] might pave the way toward the design of the PIM-polyamides (PIM-PAs) class of materials (i.e., polyamides of enhanced free volume and gas permeability). One of the earliest attempts was pursued by Weber et al. [9] who employed a spirobifluorene building block to synthesize a linear polyamide containing microporosity (Brunauer–Emmett–Teller surface area of 156 m^2^ g^−1^ was reported). The use of a Tröger’s base building block for the synthesis of linear aromatic polyamides was also reported in the literature [40]. 

**Figure 1:**
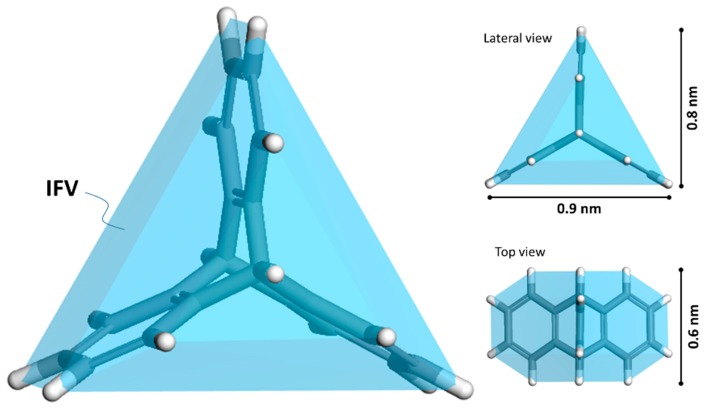
3D visual of a triptycene building block (dimensions are also provided based on molecular dynamic simulation).

Synthesis of linear polymers of high free volume can be successfully achieved by using the triptycene (Figure 1) building block [41,42,43,44,45] that is a member of the iptycene family [46], characterized by the presence of [2,2,2] bicyclic bridges linked together by three arene rings. The nonplanar conformation of the triptycene unit frustrates efficient polymer chain packing promoting the formation of free volume [47]. Moreover, this building block presents a peculiar paddle-wheel-like conformation that further improves the free volume of the polymeric matrix, due to a steric effect; in fact, triptycenes are large moieties characterized by a structurally fixed internal free volume (IFV)—as indicated in blue in Figure 1. Although triptycene-based linear polyamides were previously described in the literature [48,49,50,51,52,53,54,55], none of these studies aimed to fine-tune the properties of these polymers, in order to produce attractive combinations of gas permeability and permselectivity for industrial gas separation applications. 

**Scheme 1:**
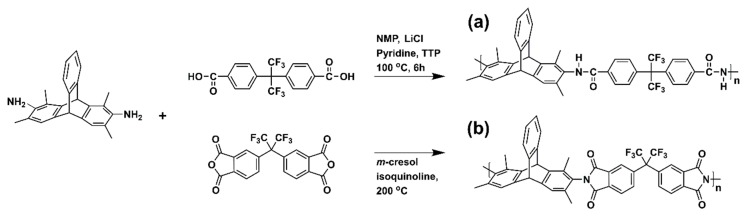
Synthesis scheme of: (**a**) 6FBBA-TMDAT polyamide and (**b**) 6FDA-TMDAT polyimide [45].

In this work, we synthesized a new, linear, and solution-processable *polyamide of intrinsic microporosity* (PIM-PA) obtained via polycondensation reaction of *o*-tetramethyldiamino-triptycene (TMDAT) with 4,4′-(hexafluoroisopropylidene)bis(benzoic acid) (6FBBA)—see Scheme 1a. The 6FBBA-TMDAT polyamide was characterized by: ^1^H NMR, Fourier transform infrared (FTIR), thermogravimetric analysis (TGA), wide-angle X-ray diffraction (WXRD), Brunauer–Emmett–Teller (BET) surface area, and molecular dynamics simulations. The pure- and mixed-gas transport properties of 6FBBA-TMDAT are reported to assess its potential for membrane-based gas separations.

A recently reported structurally related polyimide [45] derived from TMDAT and 4,4′-(hexafluoroisopropylidene)diphthalic anhydride (6FDA) is shown in Scheme 1b. Here, we demonstrated that the hydrogen-bonding effects of the amide linkages in 6FBBA-TMDAT induced significant differences in polymer packing and gas transport properties compared to 6FDA-TMDAT polyimide. 

## 2. Materials and Methods

### 2.1. Materials

Anhydrous *N*-methyl-2-pyrrolidone (NMP), *N*,*N*-dimethylacetamide (DMAC), anhydrous tetrahydrofuran (THF), pyridine, and triphenylphosphite (TPP) were purchased from Sigma Aldrich (St. Louis, MO, USA). Anhydrous lithium chloride was purchased from Sigma Aldrich and dried under vacuum at 160 °C for 10 h before use. 4,4′-(Hexafluoroisopropylidene)bis(benzoic acid) (6FBBA) was purchased from Sigma Aldrich and dried under vacuum at 80 °C before use. 1,3,6,8-Tetramethyl-2,7-diamino-triptycene (TMDAT) was prepared according to the procedure reported by Ghanem et al. [45].

### 2.2. Characterization Methods

Proton nuclear magnetic resonance spectrum (^1^H NMR) was recorded on a Bruker (Billerica, MA, USA) DRX 400 spectrometer. Chemical shifts (δ) are reported in parts per million (ppm) and referenced to tetramethylsilane. FTIR measurements were carried out with a Varian (Santa Clara, CA, USA) 610-IR FTIR spectrometer. The viscosity of a 20 wt % solution of 6FBBA-TMDAT polyamide in NMP was measured with a Brookfield (Middleboro, MA, USA) CAP 2000 cone-and-plate viscometer at 450 rpm. Wide-angle X-ray diffraction (WXRD) was performed on a Bruker D8 Advance diffractometer (Billerica, MA, USA) using a scanning rate of 1 s/step with 0.02° per step ranging from 5 to 50°. The multiple amorphous peaks in the WXRD spectrum were then deconvoluted using Peak Analyzer in Origin software (OriginLab Corporation, Northampton, MA, USA), and the average chain spacing (*d*-spacing) was calculated with Bragg’s law. The thermal degradation temperature of the polyamide was determined by thermogravimetric analysis (TGA, TA Q-5000, TA Instruments, New Castle, DE, USA) under N_2_ atmosphere at a heating rate of 3 °C min^−1^ from room temperature to 800 °C. 

A Micromeritics (Norcross, GA, USA) ASAP-2020 was employed to perform BET measurement using nitrogen sorption at 77 K. Before measurement, a 6FBBA-TMDAT powder sample was first dried at 120 °C under vacuum for 24 h. Micromeritics ASAP 2020 software (v.4.02) was used to calculate the BET surface area. CO_2_ and CH_4_ isotherms at 35 °C in the pressure range 1–15 atm were determined with a gravimetric IGA system (Hiden Isochema, Warrington, UK). 

Material Studio 2017 software (BIOVIA, San Diego, CA, USA) was used for the analysis of the torsion energy vs. rotation angle around the amide unit; we imposed: (i) the *pcff* forcefield, (ii) geometrical optimization, (iii) and ultrafine quality of estimated energies over 360 steps between −180 to 180° angles. Amorphous cell module calculations (*pcff* forcefield and a number of five polymeric chains of five repetitive units each were chosen were also pursued). We set a probe radius of the Connolly’s surface of 1.6  Å, and the software estimated the volume of the amorphous cell occupied by the polymer ($V_{occ.}$) and the remaining free volume ($V_{free}$); we used this information to calculate the FFV as: $FFV=V_{free}/\left(V_{free}+V_{occ.}\right)$.

### 2.3. Polymer Synthesis

The TMDAT diamine monomer was prepared according to a previously reported procedure in four steps starting from 1,3,6,8-tetramethyl-anthracene, initially prepared from the Friedel-Crafts alkylation reaction between *m*-xylene and dichloromethane in the presence of aluminum chloride [56]. Heating the diazonium salt of 2-aminobenzoic acid with tetramethyl-anthracene in dichloroethane yielded 1,3,6,8-tetramethyl-triptycene. Selective nitration of the tetramethyl-triptycene using potassium nitrate and trifluoroacetic anhydride followed by reduction of the dinitro groups gave the diamine monomer TMDAT [45].

**Synthesis of 6FBBA-TMDAT.** The 6FBBA-TMDAT polyamide was prepared by the Yamazaki-Higashi phosphorylation polycondensation procedure [57] between equimolar amounts of the commercially available dicarboxylic acid (6FBBA) and the TMDAT diamine monomer in anhydrous NMP as a solvent and using TPP and pyridine as condensing agents (Scheme 1). First, a mixture of TMDAT (0.24 g, 0.705 mmol), 6FBBA (0.277 g, 0.705 mmol), NMP (2 mL), LiCl (0.22 g), TPP (0.45 g, 1.45 mmol) and pyridine (0.45 mL) was added to a dry 10 mL Schlenk tube. After heating in an oil bath for six hours at 120 °C under a nitrogen atmosphere, the clear viscous solution was cooled and slowly added to methanol (200 mL) to precipitate the polymer. The resulting white fibrous polymer was collected by filtration, washed several times with methanol and hot water and then purified by re-precipitation from a DMAc/methanol mixture. Finally, drying under vacuum at 130 °C for 20 h gave a white fibrous material (90% yield). FT-IR (ν, cm^−1^): 3286 (amide N–H stretching), 3028, 3062 (aromatic C–H stretching), 2925 (aliphatic C–H stretching), 1657 (amide carbonyl stretching), 1526, 1491 (aromatic C=C ring stretching), 1249 (asymmetric C–O–C stretching), 1020 (symmetric C–O–C stretching), 1136 (C–F stretching). ^1^H NMR (DMSO-*d*_6_, 400 MHz, δ ppm): 2.08 (s, 6H), 2.36 (s, 6H), 5.51 (s, 1H), 6.08 (s, 1H), 7.00 (br. m, 2H), 7.21 (s, 2H), 7.44–7.54 (m, 6H), 8.05 (d, 4H), 9.81 (s, 2H). BET surface area = 396 m^2^ g^−1^. TGA analysis under N_2_ atmosphere: Initial weight loss, due to thermal degradation commenced at *T_d_* ~ 450 °C. Dynamic viscosity of a 20 wt % solution of 6FBBA-TMDAT in NMP was 2.68 ± 0.03 g cm^−1^ s^−1^ (three measurements). 

### 2.4. Film Preparation

6FBBA-TMDAT films were obtained from DMAc solution (5 *w*/*v* %) by slow evaporation of the solvent at 80 °C from a leveled and covered glass Petri dish under a nitrogen stream. The 6FBBA-TMDAT films were further dried at 120 °C and later soaked in methanol for 48 h, air-dried and then post-dried at 130 °C under vacuum for 24 h. The absence of residual solvent was confirmed by TGA analysis. The resulting film had a thickness of about 73 μm, which was measured by a digital micrometer (Mitutoyo (Aurora, IL, USA) 547-400S with an accuracy of 0.00381 mm). Effective permeation area values were determined from coupon scan and image analysis. The sample tested for pure-gas permeabilities was aged up to 132 days (methane permeability was also tested after 200 days) upon storage under vacuum in a desiccator. Geometrical density was determined from the average of five weight and area measurements on a fresh sample at 22 °C. 

### 2.5. Pure- and Mixed-Gas Permeation Coefficients

The constant-volume/variable-pressure technique [58] was used to observe pure-gas permeabilities of He, H_2_, N_2_, O_2_, CH_4_, and CO_2_. The same technique was also used to determine CO_2_-CH_4_ mixed-gas permeability and selectivity in 6FBBA-TMDAT up to 30 atm feed pressure, as described by O’Brien at al. [58]. For all tests, the temperature was set at 35 °C. The local slope of experimental permeate pressure vs. time was recorded to calculate pure- and mixed-gas permeabilities from:

(1) $$P_{i}=10^{10}\frac{y_{i}\cdot V_{perm}\cdot l}{x_{i}\cdot p_{feed}\cdot T\cdot R\cdot A}\cdot \frac{dp}{dt},$$

where $P_{i}$ is the gas permeability in barrer (1 barrer = 10^−10^ cm^3^ (STP) cm cm^−2^ s^−1^ cmHg^−1^), *x_i_* and *y_i_* are feed and permeate mole fractions, respectively (in the case of pure-gas permeability *x_i_* and *y_i_* are equal to one), *V_perm_* is the permeate volume, *l* is the thickness of the membrane, *p_feed_* is the feed pressure, *R* is the universal gas constant, *A* is the area of the film, and *dp*/*dt* is the local slope of permeate pressure vs. time experimental data. Compositions of the permeate mixed-gas were analyzed via an Agilent Technologies (Santa Clara, CA, USA) 3000A Micro GC. During mixed-gas permeability measurement, the ratio of permeate flow to feed flow (stage cut) was always maintained <1%. Fresh and aged film samples were always degassed in the permeation system under high vacuum for at least 24 h prior to testing.

## 3. Results and Discussion

### 3.1. Polymer Characterization

**Figure 2:**
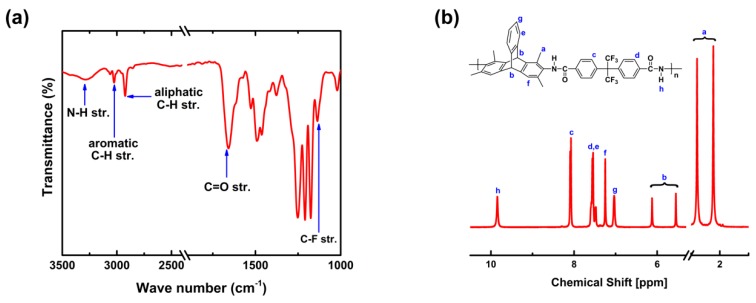
(**a**) FTIR and (**b**) ^1^H NMR spectra of 6FBBA-TMDAT polyamide.

The chemical structure of 6FBBA-TMDAT was confirmed by FTIR and ^1^H NMR spectroscopy. The FTIR spectrum (Figure 2a) showed the characteristic absorption bands of the amide group at frequencies in the range 3150–3410 cm^−1^ for –NH stretching and 1657 cm^−1^ for C=O stretching. The aliphatic and aromatic C–H stretching bands appeared at frequencies of 2925 and 3062 cm^−1^, respectively. In the ^1^H NMR spectrum of 6FBBA-TMDAT (Figure 2b), the absorption band at 9.81 ppm is assigned to the amide protons (H^h^), confirming the formation of amide linkages. The protons of the methyl groups (H^a^) appeared as two singlets at 2.08 and 2.36 ppm, whereas the bridgehead protons of the triptycene unit (H^b^) appeared at 5.51 and 6.08 ppm. The aromatic protons (H^c–g^) resonated in the range of 7.00–8.05 ppm.

**Figure 3:**
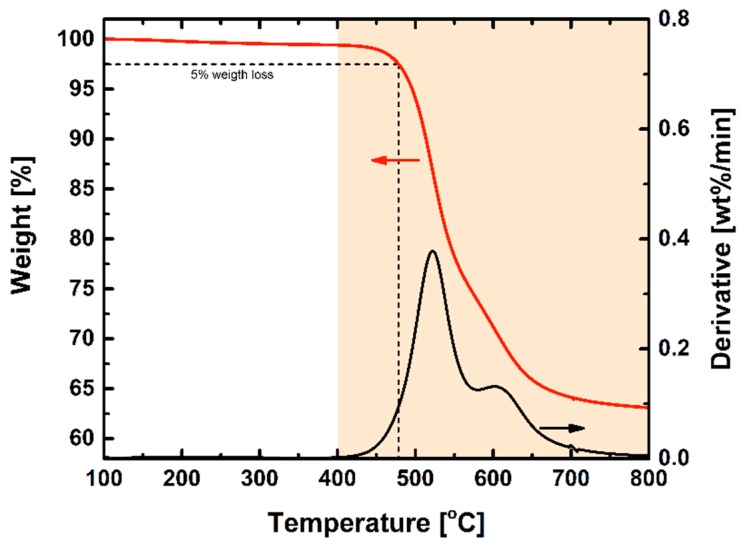
N_2_ atmosphere TGA analysis of methanol-treated 6FBBA-TMDAT polyamide film dried under vacuum at 130 °C for 24 h.

Figure 3 displays the thermogravimetric analysis (TGA) of 6FBBA-TMDAT; the absence of weight loss in the range 100–450 °C demonstrated that the methanol treatment successfully extracted all DMAc solvent from the 6FBBA-TMDAT sample. The degradation temperature at 5% weight loss (*T_d_*_,5%_) was about 480 °C (Table 1); this *T_d_*_,5%_ is comparable to that of 6FDA-TMDAT [45] (synthesized by polycondensation of 4,4′-(hexafluoroisopropylidene) diphthalic anhydride (6FDA) and TMDAT) representing the polyimide counterpart of 6FBBA-TMDAT polyamide. TGA was also employed to ensure the absence of any residual solvent in film samples before pure- and mixed-gas permeation tests.

**Figure 4:**
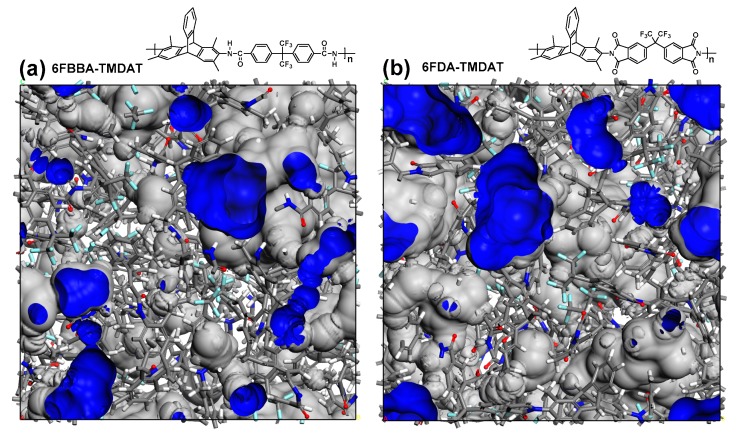
Amorphous-cell simulation of chain packing for: (**a**) 6FBBA-TMDAT polyamide and (**b**) 6FDA-TMDAT polyimide.

The *amorphous cell* module of Material Studio 2017 software was applied to simulate the space-filling ability of 6FBBA-TMDAT using the geometric density of 6FBBA-TMDAT measured in-house (1.15 ± 0.04 g/cm^3^). The same simulation procedure was also performed for 6FDA-TMDAT polyimide (see properties in Table 1). Clearly, the amorphous cell of the polyamide (Figure 4a) is more densely packed than that of 6FDA-TMDAT polyimide (Figure 4b); moreover, upon building the amorphous cell, the computer program provided occupied volume ($V_{occ.}$) vs. free volume ($V_{free}$) estimates that were used to calculate the fractional free volume (FFV) of both polymers. Accordingly, the FFV value for 6FBBA-TMDAT was 0.217 (a high value for polyamides) positioning this polyamide at the high end of the range of FFV defined by correlations of gas permeability vs. fractional free volume reported in the classical literature [15]; hence, the insertion of the TMDAT building block was very effective in enhancing the FFV. The 6FDA-TMDAT polyimide showed a higher FFV value of 0.254; therefore, we anticipated to observe a higher permeability of this polyimide when compared to the structurally related 6FBBA-TMDAT polyamide, as discussed below.

**Figure 5:**
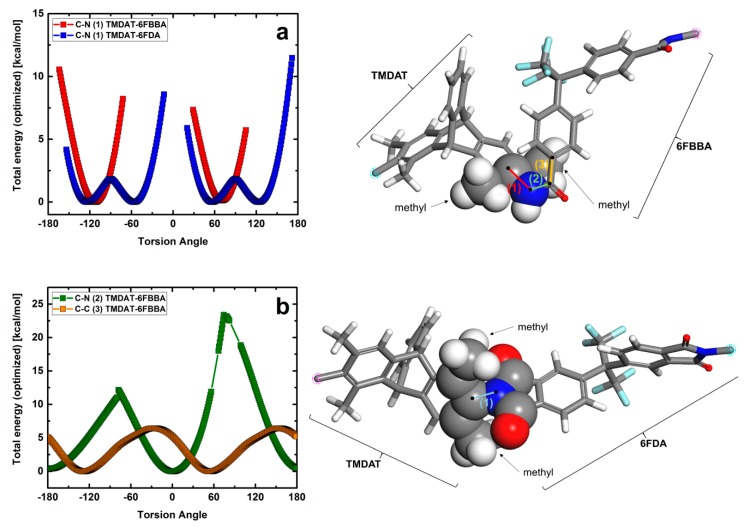
Torsion energy vs. torsion angle for the amide group of the 6FBBA-TMDAT (three dihedral angles) and for the single dihedral angle of the imide group of the 6FDA-TMDAT (polyimide). Rotation freedom around: (**a**) The C–N (1) bond of both 6FBBA-TMDAT and 6FDA-TMDAT; (**b**) for the remaining C–N (2) and C–C (3) bonds of the amide group of the polyamide.

The difference in FFV between 6FBBA-TMDAT and 6FDA-TMDAT (Table 1) reflects the chain rigidity and chain interaction properties of these polymers. Generally, FFV (and typically gas permeability) increases as the polymer exhibits increased intrachain rigidity and interchain spacing, and shows less interchain interactions (hydrogen bonding or charge transfer complex formation tend to reduce FVV). In particular, chain motion of the 6FBBA-TMDAT polyamide is described here based on a torsion analysis via computer-assisted molecular simulations (Material Studio 2017 program was used). This description was further extended by including a comparison in terms of torsion energy vs. allowed torsion angles between the 6FBBA-TMDAT polyamide and its equivalent 6FDA-TMDAT polyimide. For both polymers, Figure 5a shows the rotational freedom around the C-N bond (1) directly linked to the TMDAT unit. The methyl groups of the TMDAT unit practically lock the rotation to ~120° for the polyamide and ~180° for the polyimide; in fact, the hydrogen of the N-H group of the polyamide is located closer to the two methyl groups of the TMDAT than the oxygens of the C=O groups of the polyimide. The polyamide can still rearrange itself, due to the rotational freedom around the C–N (2) and C–C (3) bonds (Figure 5b). 

**Figure 6:**
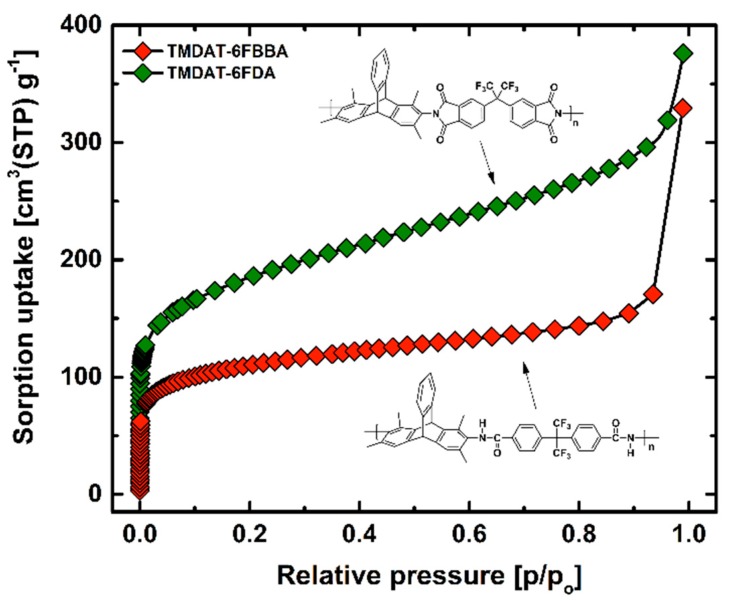
N_2_ sorption isotherms at 77 K of the 6FBBA-TMDAT polyamide and 6FDA-TMDAT polyimide [45].

Because of the higher rotational freedom (i.e., lower intrachain rigidity) around the bonds of the amide group, the 6FBBA-TMDAT polyamide can pack its polymeric chains more efficiently than the 6FDA-TMDAT polyimide. N_2_ sorption at 77 K (Figure 6) confirmed the simulation results; in fact, N_2_ uptake in the 6FDA-TMDAT polyimide was higher than in the 6FBBA-TMDAT polyamide; the BET surface area of 6FDA-TMDAT polyimide (620 m^2^ g^−1^) was ~57% higher than that of 6FBBA-TMDAT polyamide. Nevertheless, because of the presence of the tetra-substituted triptycene block, the 6FBBA-TMDAT polyamide showed a surprisingly high BET surface area of 396 m^2^ g^−1^.

**Figure 7:**
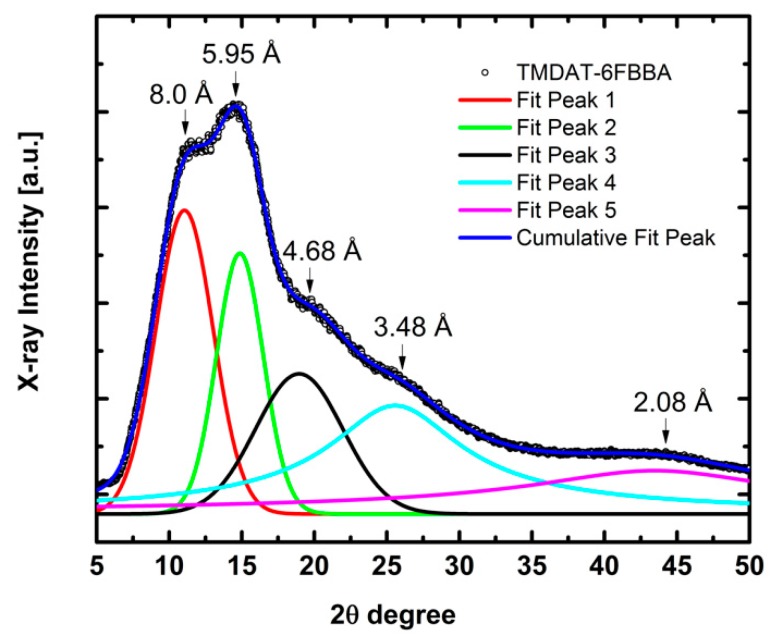
WXRD spectrum of 6FBBA-TMDAT polyamide and peaks deconvolution.

The WXRD spectrum of the 6FBBA-TMDAT polyamide is shown in Figure 7; several amorphous peaks with two overlapping amorphous halos with an average chain spacing of 8.0 and 5.95 Å, respectively, can be observed. Also, three additional peaks located around 4.7, 3.5, and 2.1 Å were identified. The two main peaks with large chain spacing derive from the introduction of the bulky triptycene building block in the polymer repeat unit. The weak amorphous peak at about 2.1 Å could be an indication of the occurrence of strong interchain interactions between the amide sites through hydrogen bonding. A similar peak was also found for the triptycene based polyimide with hydroxyl-functionalized diphenyl-hexafluoropropane unit [59], where –OH group interchain hydrogen bonding was attributed to the formation of this high-angle amorphous peak.

### 3.2. Pure-Gas Sorption, Permeation, and Physical-Aging

In this section, we describe how chemical structure, chain packing, and free volume distribution correlate with pure-gas transport properties of 6FBBA-TMDAT polyamide. 

**Figure 8:**
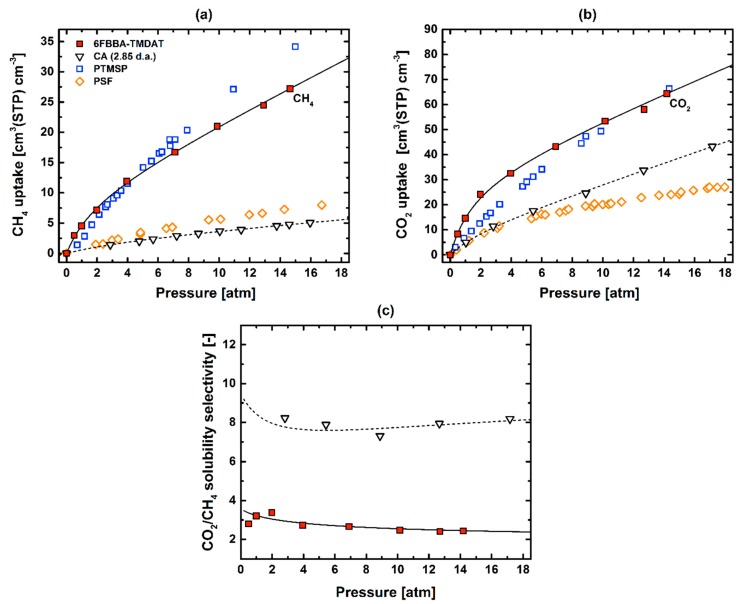
(**a**) CH_4_ and (**b**) CO_2_ pure-gas sorption isotherms vs. gas pressure. (**c**) CO_2_/CH_4_ pure-gas solubility selectivity of 6FBBA-TMDAT polyamide (red squares) and CA (black triangles) vs. gas pressure. Gas sorption data of polysulfone (PSF—orange diamonds) [62], poly[1 -trimethylsilyl-1-propyne] (PTMSP—blue squares) [60], and cellulose acetate (CA) [61] were digitalized from the literature. A dual-mode sorption model analysis [63] (not discussed) allowed to draw all prediction curves.

CH_4_ and CO_2_ uptakes of 6FBBA-TMDA are displayed in Figure 8a,b in the range of 0–15 atm at 35 °C. In comparison with a high-free-volume polymers, such as PTMSP [60] (also plotted in Figure 8a,b), at pressures lower than 5 atm, 6FBBA-TMDAT polyamide displayed higher CH_4_ and CO_2_ uptakes possibly reflecting a higher content of microporosity and ultra-microporosity—note: to reduce surface energy, gas molecules tend to sorb in small pores first. On the contrary, at pressures higher than ~7 atm for CH_4_ and ~14 atm for CO_2_, gas sorption in PTMSP is higher than in 6FBBA-TMDAT, and this behavior agrees with the fact that PTMSP is a polymer with pore size distribution shifted towards larger micropores, i.e., in the range of ~10–20 Å [41]. Figure 8a,b also reveal that CO_2_ and CH_4_ uptakes in 6FBBA-TMDAT were significantly higher than in the case of low-free-volume glassy cellulose acetate (CA) [61] and polysulfone (PSF) [62] which are the most commonly used commercial gas separation materials. Interestingly, a comparison between the 6FBBA-TMDAT polyamide and CA revealed a greater CO_2_/CH_4_ solubility selectivity of commercial CA (Figure 8c). Note: the solubility selectivity at infinite dilution of CA was 2.7 times higher than that of 6FBBA-TMDAT. 

The discussion is now extended to the pure-gas permeability properties of 6FBBA-TMDAT polyamide at 35 °C. He, H_2_, N_2_, O_2_, CH_4_, and CO_2_ pure-gas permeabilities measured at 2 atm are listed in Table 2. 6FBBA-TMDTA aged for two days (‘*fresh*’ sample) exhibited noteworthy gas permeabilities, e.g., 226, 144, and 33 barrer for H_2_, CO_2_, and O_2_, respectively. Pure-gas permeability of 6FBBA-TMDAT increased in the following order: H_2_ > He > CO_2_ > O_2_ > N_2_ > CH_4_. The fresh 6FBBA-TMDAT polyamide film had moderately high permselectivities, e.g., 39, 25, and 4.7 for H_2_/CH_4_, CO_2_/CH_4_ and O_2_/N_2_, respectively.

**Figure 9:**
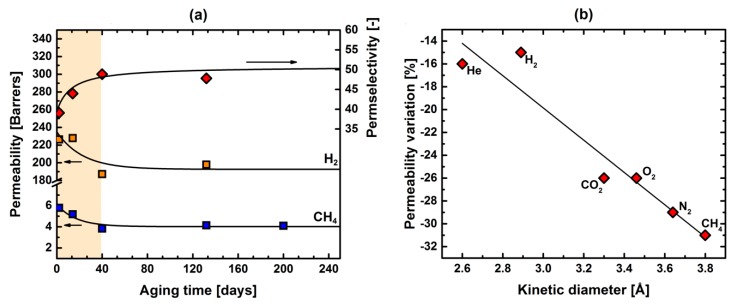
(**a**) Pure-gas permeability and permselectivity of 6FBBA-TMDAT polyamide vs. aging time (H_2_–CH_4_ gas pair); (**b**) aging-induced permeability variation (calculated at the aging knee) vs. kinetic-gas diameter. Lines are drawn to guide the eye.

Because extensive hydrogen bonding induces tight interchain packing and discourages chains mobility, conventional low-free-volume polyamides typically do not display significant physical aging tendency. However, in view of the high surface area exhibited by 6FBBA-TMDAT, physical aging induced some reduction of 6FBBA-TMDAT permeability (Table 2). The aging knee—i.e., the starting point of the region of *quasi-steady-state* permeability [44]—of 6FBBA-TMDAT appeared after ~40 days. For example, for the H_2_–CH_4_ gas pair, this knee can be identified at the right boundary of the highlighted region of Figure 9a; CH_4_ and H_2_ permeabilities decreased by 30 and 15% respectively, whereas H_2_/CH_4_ permselectivity increased by 20% from the fresh sample values. A general analysis of all studied gases revealed that the reduction of permeability (at the aging knee) from fresh sample values approximately followed a linear trend of the kinetic gas diameters (see Figure 9b).

**Figure 10:**
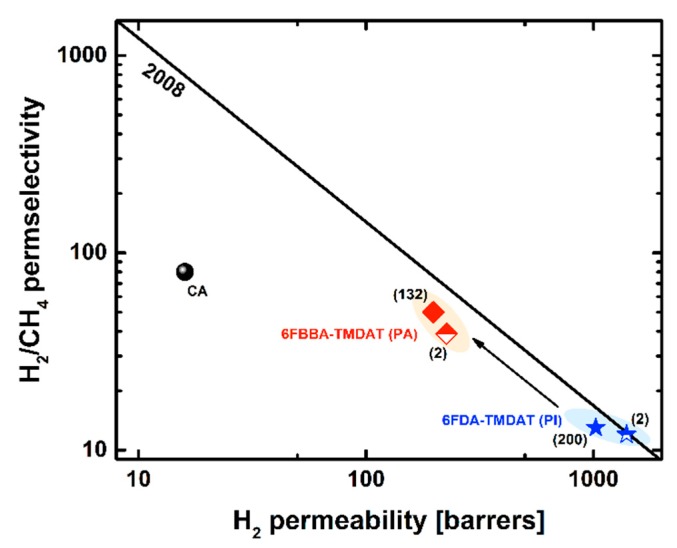
Location of 6FBBA-TMDAT polyamide on pure-gas H_2_-CH_4_ upper bound (2008) plot [65]. Cellulose acetate (CA) [64] and 6FDA-TMDAT [45] are shown for comparison. Aging (closed symbols) was recorded for 132 and 200 days for 6FBBA-TMDAT (polyamide) and 6FDA-TMDAT (polyimide), respectively (as also indicated in the plot).

In Table 2, pure-gas permeability and permselectivity of 6FBBA-TMDAT polyamide are also compared with the structurally related 6FDA-TMDAT polyimide [45] and conventional cellulose acetate (CA, with a degree of acetylation of about 2.9 [64]). The 6FDA-TMDAT polyimide is much more permeable than 6FBBA-TMDAT polyamide because of the higher surface area (Figure 6), FFV, and chemical bond rotation freedom as discussed earlier in this work (Figure 4 and Figure 5). For example, aged films samples of 6FBBA-TMDAT polyamide and 6FDA-TMDAT polyimide exhibited CO_2_ permeabilities of 109 and 1150 barrer, respectively. On the other hand, hydrogen bonding ensured higher gas-pair permselectivities for 6FBBA-TMDAT polyamide than 6FDA-TMDAT polyimide (Table 2); for example, pure-gas O_2_/N_2_ and CO_2_/CH_4_ selectivities (4.8 and 26) of aged 6FBBA-TMDA were more than ~30% and ~70% higher than those of its polyimide counterpart 6FDA-TMDAT (3.7 and 15), respectively. Likewise, H_2_/CH_4_ and H_2_/N_2_ pure-gas permselectivity of 6FBBA-TMDA was 3.8 and 2.3 fold greater than 6FDA-TMDAT (at the aging knee), respectively, as shown in Figure 10 and Table 2. The good combination of both permeability and permselectivity of 6FBBA-TMDAT in both fresh and aged samples placed it close to the 2008 H_2_/CH_4_ (Figure 10) and H_2_/N_2_ (not shown) upper bounds.

Compared to conventional low-free-volume glassy polymers, such as CA, 6FBBA-TMDAT demonstrated (Table 2) much higher permeability (e.g., ~9 and ~16 times higher for H_2_ and CO_2_ permeability, respectively) because of both higher solubility and diffusion coefficients (see Table 3 for the CO_2_–CH_4_ pure-gas pair at 2 atm and 35 °C). However, enhancement in permeability coupled with a decrease in permselectivity followed the traditional permeability/selectivity trade-off relationship. As a result, CA is generally more permselective than 6FBBA-TMDAT (Table 2). For the CO_2_–CH_4_ pure-gas system, the data in Table 3 indicate that the permselectivity in CA benefitted from the above mentioned solubility selectivity advantage (Figure 8c); whereas 6FBBA-TMDAT polyamide fills this solubility selectivity gap by a superior diffusion selectivity (Table 2).

### 3.3. CO_2_–CH_4_ Mixed-Gas Permeation

**Figure 11:**
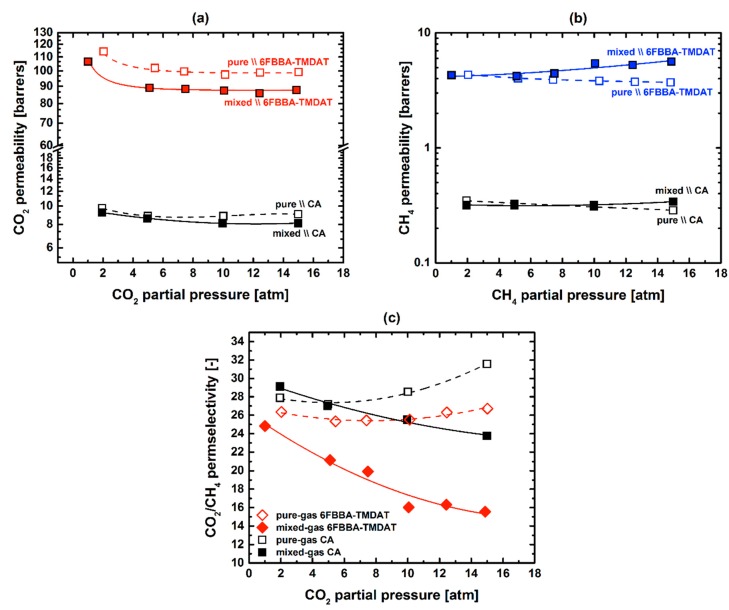
Experimental (**a**) CO_2_ and (**b**) CH_4_ pure- and mixed-gas permeability vs. partial gas pressure for 6FBBA-TMDAT polyamide and CA [64]. (**c**) pure- and mixed-gas CO_2_/CH_4_ permselectivity of the same polymers. Lines are drawn to guide the eye.

The 6FBBA-TMDAT film was also tested for mixed-gas permeation of a 50:50 mol% CO_2_:CH_4_ mixture up to 30 atm total feed pressure (at 35 °C). As shown in Figure 11a, CO_2_ pure-gas permeability decreased with increasing pressure, due to the saturation of Langmuir’s sites. On the other hand, CO_2_–CH_4_ competitive sorption was responsible for the decrease of CO_2_ mixed-gas permeability from the pure-gas values (Figure 11a). In the range of studied pressures, both pure- and mixed-gas CO_2_ permeability isotherms did not reveal a minimum and the subsequent positive slope that generally marks the occurrence of *plasticization*. In other words, the occurance of plasticization hides behind the effects on gas permeability of saturation of Langmuir’s sorption sites and competitive sorption. Although in a different range of permeability, pure- and mixed-gas CO_2_ permeability of 6FBBA-TMDAT followed the general behavior trends of CA [64] (Figure 11a).

For both 6FBBA-TMDAT and CA, pure-gas CH_4_ permeability decreased slightly with pressure, due to Langmuir’s sites saturation (Figure 11b). Contrarily to the just described permeation behavior of CO_2_ in the mixture, CH_4_ mixed-gas permeability of both 6FBBA-TMDAT and CA *increased* with partial pressure. Experimental studies on CO_2_–CH_4_ pure- and mixed-gas permeability, sorption and diffusion in glassy polyimide and rubbery polydimethylsiloxane membranes [66,67] showed that this CH_4_ permeability enhancement is directly correlated to CO_2_ sorption; i.e., increasing CO_2_ content in the polymer matrix activates chain mobility thus boosting CH_4_ diffusion coefficient. Hence, the occurrence of this phenomenon explains the decrease in CO_2_/CH_4_ permselectivity under mixed-gas conditions in both 6FBBA-TMDAT and CA (Figure 11c).

Finally, it is noteworthy that at 10 atm partial CO_2_ pressure (the typical well-head pressure of interest for natural gas applications [68]), the increase in mixed-gas CH_4_ permeability from the corresponding pure-gas values was 2% and 43% for CA and 6FBBA-TMDAT, respectively. Consequently, 6FBBA-TMDAT suffered a considerable loss in CO_2_/CH_4_ mixed-gas permselectivity compared to CA. This is most likely, due to the fact that the hydrogen-bonding ability of 6FBBA-TMDAT was sterically reduced by the presence of the bulky triptycene building block (Figure 1).

## 4. Conclusions

In this study, *ortho*-methyl tetra-substituted triptycene diamine (TMDAT) was successfully reacted with 4,4′-(hexafluoroisopropylidene)bis(benzoic acid) (6FBBA) to form 6FBBA-TMDAT polyamide. The polymer was soluble in aprotic solvents, such as DMAc, and exhibited excellent thermal stability (~480 °C). The BET surface area of the aromatic polyamide was 396 m^2^ g^−1^ confirming that it is an example of a polyamide of intrinsic microporosity (PIM-PA). 6FBBA-TMDAT displayed high pure-gas permeabilities and moderate permselectivities, which placed it close to the 2008 H_2_/CH_4_ and H_2_/N_2_ upper bounds. Compared to its polyimide counterpart 6FDA-TMDAT, 6FBBA-TMDAT exhibited a tighter structure and therefore improved permselectivities toward various gas pairs at cost of lower permeability. Because in 6FBBA-TMDAT the bulky triptycene unit ‘protects’ the amide H-bonding sites, some degree of physical aging was observed. The same effect was also accountable for the lower plasticization resistance of 6FBBA-TMDAT than conventional cellulose acetate used for membrane-based CO_2_/CH_4_ separation. The mixed-gas CO_2_/CH_4_ permselectivity of 6FBBA-TMDAT at 35 °C was depressed, due to the increase of CH_4_ mixed-gas diffusion associated with CO_2_ sorption.

## Figures and Tables

**Table 1:** Physical properties of 6FBBA-TMDAT polyamide. Degradation temperature at 5% weight loss (*T_d_*_,5%_), film density (ρ), surface area (S_BET_), and fractional free volume (FFV). (*) Data reported for 6FDA-TMDAT polyimide by Ghanem et al. [45]. Fractional free volume (FFV) was calculated for both polymers via molecular modeling (i.e., amorphous cell study in Material Studio 2017 software).

Polymer	*T_d_*_,5%_ [°C]	S_BET_ [m^2^ g^−1^]	ρ [g cm^−3^]	FFV [–]
6FBBA-TMDAT [this work]	480	396	1.15 ± 0.04	0.217
6FDA-TMDAT [45]	470 *	620 *	1.13 ± 0.04	0.254

**Table 2:** Aging time [days], permeability [barrer], and permselectivity of 6FBBA-TMDAT polyamide and 6FDA-TMDAT polyimide [45] films for pure gases at 35 °C and 2 atm; the conventional commercial cellulose acetate (CA) [61] is also listed for comparison.

	Aging	He	H_2_	N_2_	O_2_	CH_4_	CO_2_	H_2_/CH_4_	O_2_/N_2_	CO_2_/CH_4_
Polymer	[days]	Pure-Gas Permeability [barrer]						Ideal Selectivity [–]		
6FBBA-TMDAT [This work]	2	185	226	7.1	33	5.8	144	39	4.7	25
6FBBA-TMDAT [This work]	14	178	228	6.4	30	5.2	133	44	4.8	26
6FBBA-TMDAT [This work]	40	150	187	4.8	24	3.8	105	49	4.9	27
6FBBA-TMDAT [This work]	132	160	198	5.2	25	4.1	109	48	4.8	26
6FDA-TMDAT [45]	fresh	758	1400	113	374	121	1727	12	3.3	14
6FDA-TMDAT [45]	200	601	1024	70	261	76	1150	13	3.7	15
CA (D.S. 2.84) [61]	fresh	20	16	0.2	1.5	0.2	6.6	80	6.3	33

**Table 3:** CO_2_ and CH_4_ pure-gas solubility (S, measured gravimetrically) and diffusion coefficients (D), together with CO_2_/CH_4_ solubility selectivity, and CO_2_/CH_4_ diffusivity selectivity (2 atm and 35 °C) for 6FBBA-TMDAT polyamide and CA [64]. Diffusion coefficients were calculated from D=P/S.

	S, CH_4_	S, CO_2_	D, CH_4_	D, CO_2_	α_S_	α_D_
Polymer	[cm^3^(STP) cm^−3^ atm^−1^]		[cm^2^ s^−1^]		[–]	[–]
6FBBA-TMDAT [This work]	3.6	12.2	9.1 × 10^−9^	7.1 × 10^−8^	3.4	7.8
CA (D.S. 2.84) [61]	0.5	4.2	5.1 × 10^−9^	1.8 × 10^−8^	8.1	3.5
